# Longitudinal analysis of antimicrobial consumption and economic outcomes of surgical prophylaxis stewardship in a wartime Ukrainian maternity hospital

**DOI:** 10.1017/ash.2026.10349

**Published:** 2026-04-01

**Authors:** Oleksiy Pechak, Kateryna Bielka, Danylo Yevstifeiev, Lesya Yanitska

**Affiliations:** 1 Department of Medical Biochemistry and Molecular Biology, Bogomolets National Medical University, Kyiv, Ukraine; 2 Department of Surgery, Anesthesiology and Intensive Care of Postgraduate Education, Bogomolets National Medical University, Kyiv, Ukraine

## Abstract

**Objective:**

To analyze the dynamics of antimicrobial consumption and evaluate the clinical and economic outcomes of a comprehensive antimicrobial stewardship (AMS) program in a Ukrainian maternity hospital.

**Design:**

Retrospective observational study.

**Methods:**

The study was conducted at a tertiary referral maternity hospital in Kyiv, Ukraine, using two datasets. First, a longitudinal analysis of pharmacy dispensing records (2020–2024) was performed to assess consumption trends. Second, a comparative cohort analysis of 320 women undergoing gynecological surgery (preAMS [2018] vs postAMS [2023]; *n* = 160 per group) was conducted to evaluate the impact of stewardship interventions. The AMS program, implemented in 2020, utilized administrative “stop-orders” for postoperative prophylaxis and pharmacy gatekeeping for broad-spectrum agents. Economic outcomes were assessed using a patient-level micro-costing approach.

**Results:**

A profound structural shift occurred: consumption of ceftriaxone decreased from 72.4% of total prophylactic units in 2020 to 1.4% in 2024, replaced by cefazolin (0% to 44.0%). In the clinical cohorts, unjustified postoperative antibiotic use was eliminated (46.3% in 2018 to 0% in 2023; *P* < .001). The transition to single-dose prophylaxis reduced the mean direct antibiotic cost by 78.4% and coincided with a decrease in the mean length of stay from 9.85 to 4.68 days (*P* < .001). The program generated an estimated net saving of approximately $1,040 per surgical case.

**Conclusions:**

Institution-level stewardship interventions successfully shifted prescribing toward guideline-concordant use and generated substantial cost savings. This study demonstrates the resilience and feasibility of low-cost stewardship models in high-pressure, transitional healthcare environments.

## Introduction

The global rise of antimicrobial resistance (AMR) represents a critical threat to modern medicine, undermining the efficacy of essential treatments and increasing patient morbidity, mortality, and healthcare costs. Projections estimate that without effective action, AMR could cause up to 10 million deaths annually by 2050.^1^ Antimicrobial stewardship (AMS) programs have emerged as a cornerstone of the global strategy to combat AMR, with the monitoring of antimicrobial consumption serving as both a key performance indicator and a vital tool for identifying targets for improvement.^2^

A primary objective of many hospital AMS programs is the reduction of broad-spectrum antibiotics that exert significant selective pressure. Third-generation cephalosporins (3GCs), particularly ceftriaxone, are frequently identified as high-priority targets due to their association with “collateral damage,” including the disruption of the host microbiome and the selection of multidrug-resistant organisms, such as extended-spectrum beta-lactamase (ESBL)-producing Enterobacterales.^3–5^

In Ukraine, the transition to evidence-based prescribing faces unique challenges. Historical prescribing habits, characterized by a reliance on broad-spectrum agents for “defensive” prophylaxis, remain prevalent. Point prevalence surveys have revealed that WHO “Watch” group antibiotics can constitute over 90% of total consumption in some Ukrainian settings.^6,7^ This tendency, mirrored across Eastern Europe,^8^ contributes to alarmingly high resistance rates. Recent data indicate that 100% of Klebsiella pneumoniae isolates from healthcare-associated infections in Ukraine were resistant to 3GCs.^9^ This crisis has been critically exacerbated by the ongoing full-scale war, which has created a “perfect storm” for AMR through supply chain disruptions, high trauma volumes, and the overarching stress on healthcare infrastructure.^10^

Against this challenging backdrop, our institution launched a comprehensive AMS program in 2020. This study aims to analyze the dynamics of antimicrobial consumption at a large urban maternity hospital over a five-year period (2020–2024). Furthermore, utilizing a patient-level micro-costing approach, we seek to evaluate the clinical and economic outcomes of these interventions. By examining these trends, we provide a model of clinical resilience, demonstrating how effective stewardship can be maintained even within a high-pressure, resource-constrained environment.

## Methods

We conducted a retrospective analysis using two distinct datasets from a tertiary maternity hospital in Kyiv, Ukraine. First, to assess longitudinal consumption trends, we extracted hospitalwide dispensing data (encompassing all surgical and non-surgical departments) from the central pharmacy (2020–2024). This data set captured the total annual units dispensed per agent. Second, to evaluate the clinical and economic outcomes of the antimicrobial stewardship program, we performed a retrospective comparative cohort analysis of individual patient records. The study protocol was reviewed and approved by the Bioethics and Ethics Committee for Scientific Research of the Bogomolets National Medical University (Kyiv, Ukraine) (protocol No. 196, approval date: 23 June 2025). The study was conducted using anonymized data. Due to the retrospective nature of the study and the use of non-identifiable data, individual informed consent was waived.

The clinical cohort analysis compared two distinct periods: the preAMS period (2018) and the postAMS period (2023). We included 160 consecutive women per year (*n* = 320 total) who underwent gynecological surgical procedures (hysteroscopy, laparoscopy, or laparotomy). Data were extracted from electronic medical records, anesthesia charts, and nursing administration logs. The 2018 cohort reflected practice under the now-repealed Ministry of Health (MOH) Order No. 502,^11^ which permitted prolonged prophylaxis (3–5 d) and the use of third-generation cephalosporins. The 2023 cohort reflected practice under the new MOH Order No. 822,^12^ which mandates single-dose prophylaxis with first-generation cephalosporins. The use of complete annual cohorts minimized the potential impact of seasonal variation and selection bias.

The study period spanned a critical regulatory and institutional transition defined by three distinct phases. During the PreAMS period, there were no institutional restrictions on antibiotic prescribing. In 2020, our institution proactively implemented a local stewardship bundle ahead of national reforms. Key interventions included administrative “stop-orders”; (anesthesia protocols were modified to include a mandatory “stop” for prophylactic antibiotics immediately after wound closure. Continuation of antibiotics required a documented infectious indication and a separate prescription); pharmacy dispensing controls (a “gatekeeping” policy was introduced, restricting the dispensing of ceftriaxone and cefepime for surgical prophylaxis without specific justification (eg, documented allergy or colonization)); prospective audit and feedback (monthly compliance audits were initiated, with feedback provided directly to surgical teams regarding timing and choice of agents). In 2022, Ministry of Health Order No. 822 harmonized national standards with EU guidelines. It explicitly prohibited routine prophylactic use of third-generation cephalosporins, mandating single-dose cefazolin (with specific adjuvant alternatives like azithromycin or metronidazole for complex cases) and forbidding postoperative continuation. Our AMS program utilized this mandate to reinforce local interventions.

Antimicrobial agents were classified according to the Anatomical Therapeutic Chemical (ATC) classification system. For rational-use analysis, agents were categorized by spectrum of activity and according to the WHO AWaRe classification (Access, Watch, Reserve) [10]. The primary metric for longitudinal consumption was the total number of units dispensed annually. Although Defined Daily Doses (DDD) per patient-days represent the international standard, retrospective linkage of patient-days to pharmacy dispensing data was not feasible. However, the hospital’s annual surgical volume and case mix remained stable throughout the study period, rendering raw dispensing units a reliable internal proxy for structural shifts in consumption.

We conducted a retrospective micro-costing analysis from the hospital provider’s perspective. To eliminate the confounding effects of inflation and currency fluctuations over the 6-year period, we employed a “modelled cost” approach. Actual antibiotic regimens (including molecule, dose, frequency, and duration) were extracted for every patient in the 2018 and 2023 cohorts from the clinical data set. This included both standard prophylaxis and alternative regimens (eg, combinations with gentamicin, azithromycin, or doxycycline) used in complex cases. Economic inputs were derived from the National Price Catalogue of Medicines (December 2025 update), which serves as the statutory source for reference pricing and reimbursement in Ukraine.^13^ This centralized database ensures the external validity of the cost estimates.

Prices were converted to U.S. Dollars (USD) at the official National Bank of Ukraine exchange rate for December 2025 (42.5 UAH/USD). Key reference prices included ceftriaxone ($.67/1.0 g), cefazolin ($.53/1.0 g), and metronidazole ($.80/100 ml). Consumables were estimated at $.35 per administration. The cost of a surgical bed-day was estimated at $200 USD. This Figure was derived from WHO-CHOICE estimates for Ukraine, adjusted for 2025 inflation using the national Consumer Price Index, and represents a conservative average for specialized surgical care (which incorporates overhead, personnel, and indirect costs but excludes specific drugs). The total economic impact per patient was calculated as the sum of direct antimicrobial costs and hospitalization costs based on the individual length of stay (LOS).

Statistical analyses were performed using R software (version 4.3.1). Descriptive statistics summarized consumption trends. Changes in the consumption structure (2020 vs 2024) were assessed using a chi-squared test. For the patient cohorts (2018 vs 2023), differences in categorical variables (eg, postoperative antibiotic use) were assessed using Fisher’s exact test, and differences in continuous variables (eg, costs, LOS) were evaluated using the Mann-Whitney *U* test. A *P* value < .05 was considered statistically significant.

## Results

### General consumption trends, structural changes, and rational use metrics

**Figure 1.**
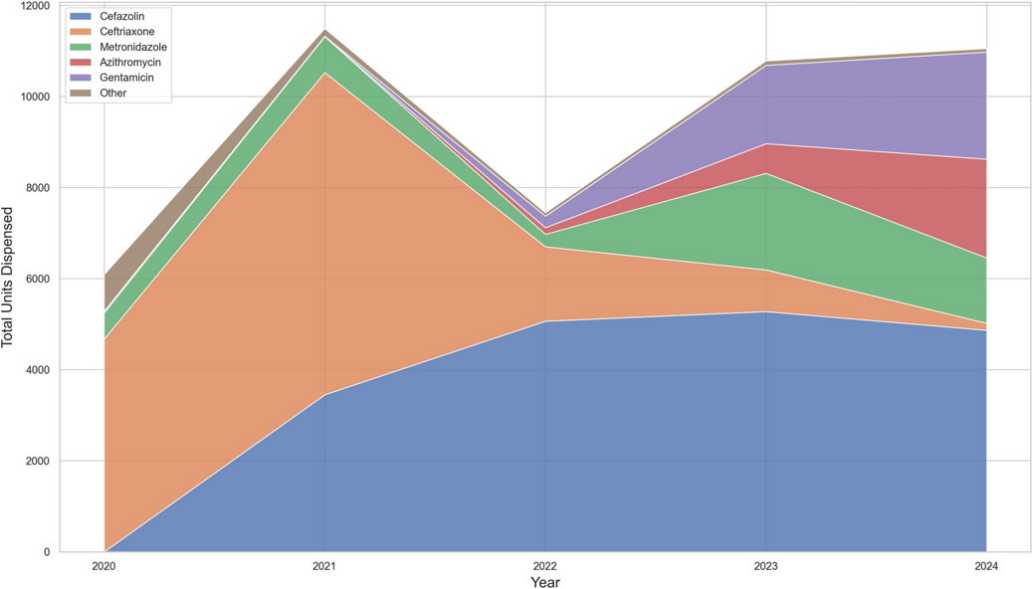
Dynamics of key antimicrobial consumption (2020–2024). The stacked area chart illustrates annual changes in the total number of hospitalwide units dispensed for the primary antimicrobial agents. While the dramatic inversion of ceftriaxone and cefazolin primarily reflects the successful stewardship intervention in surgical prophylaxis, the increases in azithromycin, gentamicin, and metronidazole represent a multifactorial trend. These increases reflect both their new protocol-mandated roles as prophylactic adjuvants for complex cases (e.g., premature rupture of membranes, severe beta-lactam allergies, instrumental deliveries) under MOH Order No. 822 and their broader therapeutic use across non-surgical departments for treating concurrent respiratory and intracellular pelvic infections during the COVID-19 pandemic and wartime sheltering conditions.

The implementation of the antimicrobial stewardship program precipitated a fundamental restructuring of institutional prescribing patterns over the five-year observation period. A chi-squared test comparing the proportional distribution of all antimicrobial agents included in the pharmacy dispensing data set between 2020 and 2024 confirmed this structural shift (*χ* ^2^ = 14,533.4; df = 12; *P* < .001). Total antimicrobial consumption fluctuated, with the lowest volume observed in 2020 (6,089 units) and the highest in 2021 (11,481 units). However, these fluctuations must be interpreted in the context of extreme wartime and pandemic-related variations in hospital throughput. As detailed in Table 1, the overall clinical workload (total medical services provided) varied dramatically, surging from 46,108 in 2020 to 96,842 in 2024. Analysis of the aggregated pharmacy dispensing data revealed a complete inversion of the dominant prophylactic agents, a structural change clearly illustrated by the longitudinal dynamics of individual agents (Figure 1). In 2020, third-generation cephalosporins constituted the cornerstone of surgical prophylaxis, with ceftriaxone alone accounting for 72.4% of total antimicrobial units dispensed. By 2024, the consumption of ceftriaxone had declined precipitously to 1.4%, effectively eliminating it from routine prophylactic use (Table 2). This broad-spectrum reliance was supplanted by first-generation cephalosporins; consumption of cefazolin rose from a negligible baseline of 0% in 2020 to become the predominant agent, representing 44.0% of all dispensed units by 2024.

**Table 1.** Annual hospital volumes and antimicrobial consumption (2020–2024)

Year	Total units dispensed	Inpatient surgical procedures*	Deliveries	Total medical services provided**
2020	6,089	1,035	2,696	46,108
2021	11,481	1,381	5,290	76,615
2022	7,449	1,106	3,419	55,793
2023	10,761	1,538	4,094	47,894
2024	11,053	1,167	5,865	96,842

*Includes one-day surgeries.

**Includes all inpatient, outpatient, diagnostic, and ambulatory services provided by the hospital.

**Table 2.** Annual consumption (Units) of the main antimicrobial agents, 2020–2024

Antimicrobial agent	2020	2021	2022	2023	2024
Ceftriaxone	4,672	7,074	1,634	916	153
Cefazolin	0	3,451	5,066	5,277	4,867
Metronidazole	574	790	274	2,120	1,434
Gentamicin	38	10	253	1,720	2,351
Azithromycin	0	0	146	651	2,170
Cefepime	568	24	0	0	0
Amikacin	194	20	40	0	0
Vancomycin	41	64	21	42	0
Ciprofloxacin	2	48	15	35	78
Imipenem	0	10	0	20	0

**Figure 2.**
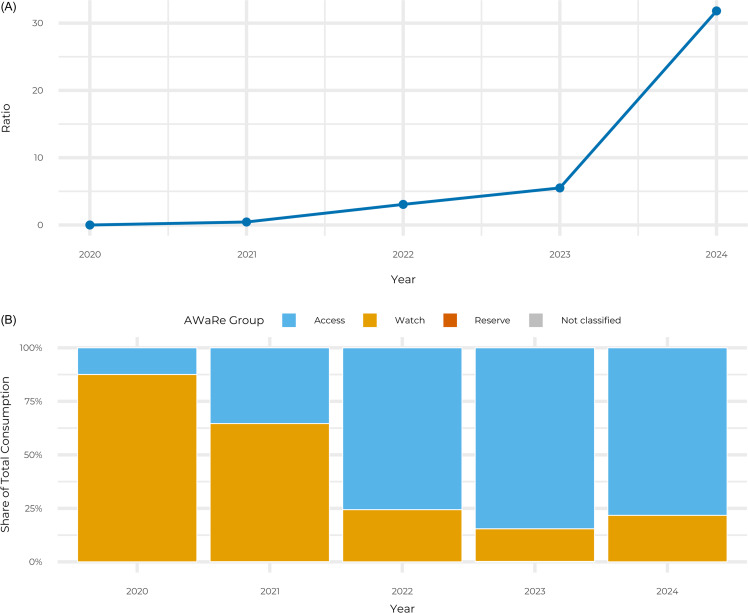
Rational use metrics (2020–2024). (A) the change in the narrow-to-broad spectrum consumption ratio. (B) the corresponding shift in the percentage share of total consumption by WHO AWaRe classification (Access, watch, reserve).

This transition resulted in a profound improvement in rational use metrics (Figure 2). The narrow-to-broad spectrum consumption ratio, which stood at 0 in 2020 due to the near-total absence of narrow-spectrum agents, ascended to 31.8 by the end of the study period (Figure 2A). Concurrently, the proportion of antibiotics classified within the WHO AWaRe “Access” group surged from 12.5% in 2020 to a peak of 84.6% in 2023, substantially exceeding the WHO target threshold of 60% (Figure 2B). Conversely, reliance on the “Watch” group agents diminished from 87.5% to 21.7%, reflecting a sustained adherence to stewardship protocols despite the external pressures of the wartime environment.

### Clinical efficiency and economic outcomes

Analysis of the patient-level cohorts (*n* = 160 per group) revealed significant improvements in clinical outcomes following implementation of the AMS program. The practice of unjustified postoperative antibiotic prophylaxis was eliminated. In 2018, 46.25% (74 of 160) of surgical patients received postoperative antibiotics, whereas in 2023, no patients received postoperative antibiotics (0 of 160). This reduction was highly statistically significant (Fisher’s exact test, *P* < .001).

Micro-costing analysis revealed that the transition to guideline-concordant prophylaxis reduced the mean direct antibiotic cost per surgical case by 78.4%. In the preAMS period, the mean cost was $8.16 (SD 9.51) per patient, driven largely by the cumulative cost of multi-day regimens comprising ceftriaxone and metronidazole. In the postAMS period, the mean cost decreased to $1.76 (SD 1.12) per patient. Notably, this 2023 cost reflects a realistic case mix: while 56% of patients received standard cefazolin monotherapy (theoretical cost $1.41), the remaining 44% required alternative regimens due to allergies or high-risk indications, including combinations with gentamicin (19%), azithromycin (16%), or doxycycline (8%). Despite the inclusion of these combination therapies, the total pharmaceutical burden remained significantly lower than baseline due to the cessation of postoperative dosing.

However, the most significant economic benefit was derived from the optimization of hospital resource utilization. The rationalization of antibiotic therapy was associated with a significant reduction in the mean length of stay, which decreased from 9.85 days (SD 9.50) in 2018 to 4.68 days (SD 4.33) in 2023 (Mann-Whitney *U* test, *P* < .001). When factoring in the reduction in bed-day utilization ($1,034 saving per patient) alongside medication savings ($6.40 per patient), the stewardship program generated an estimated total net saving of approximately $1,040 per surgical case. These findings indicate that the administrative restriction of broad-spectrum agents and the enforcement of single-dose protocols not only improved guideline concordance but also yielded substantial cost-minimization advantages for the healthcare provider.

## Discussion

The observed transformation in antimicrobial consumption reflects a successful shift from defensive, empirical prescribing to a standardized, evidence-based model. The near-total replacement of ceftriaxone with cefazolin demonstrates that guideline adherence can be achieved even in healthcare cultures traditionally reliant on broad-spectrum coverage. This finding aligns with international data confirming the non-inferiority of first-generation cephalosporins for surgical prophylaxis, which offer the critical advantage of reducing selective pressure on the hospital microbiome.^14–16^ Our institutional reduction in third-generation cephalosporin use mirrors successes reported in other resource-limited settings,^17^ yet the magnitude of the reduction, from 72.4% to 1.4%, suggests that administrative “forcing functions,” such as stop orders and dispensing restrictions, may be more effective than educational interventions alone in driving rapid behavioral change.^18^

The analysis highlights the dynamic complexity of stewardship implementation. The emergence of gentamicin, azithromycin, and metronidazole as significant prophylactic agents illustrates the “squeezing the balloon” phenomenon, where restricting one class necessitates alternatives.^19^ However, this shift directly reflects strict adherence to the updated national standard (MOH Order No. 822). According to this mandate, intravenous metronidazole is now required for instrumental vaginal deliveries, while gentamicin serves as the designated alternative for severe beta-lactam allergies, a subgroup historically managed inappropriately with ceftriaxone.^20^ Similarly, the sharp rise in azithromycin is protocol-driven, mandated as an adjuvant for Cesarean sections complicated by premature rupture of membranes and for surgical abortions. While this diversification represents a rational, guideline-concordant adaptation rather than chaotic empirical prescribing, the resulting surge in macrolide consumption warrants careful scrutiny. Their widespread use introduces new resistance risks.^21^ Therefore, future stewardship cycles must rigorously audit these combination regimens to ensure they remain strictly targeted and do not evolve into a new form of empirical over-coverage.

The micro-costing analysis provides a nuanced understanding of the economic value of stewardship. Contrary to the common assumption that savings are derived solely from lower drug acquisition costs, our data show that the unit cost of guideline-concordant prophylaxis in certain cases (high-dose cefazolin plus necessary combinations) was marginally higher than the historical baseline. The substantial net saving ($1,040/patient) was primarily driven by improved hospital throughput.^22^ By eliminating multi-day postoperative dosing, the program removed a significant logistical barrier to discharge. However, we acknowledge this 50% LOS reduction is multifactorial. As demonstrated in our companion mathematical modeling of this cohort,^23^ overarching secular trends, such as improved sterile techniques, administrative efficiency, and rigorous hygiene measures introduced during the COVID-19 pandemic, were the dominant predictors of earlier discharge. The cessation of mandatory prolonged antibiotics served as a critical enabling factor for these rapid-discharge pathways, optimizing clinical efficiency alongside direct pharmacy savings.^24^

These findings have broader implications for healthcare systems operating under extreme pressure. The study demonstrates that low-cost, protocol-based interventions can yield high-impact results without the need for expensive digital infrastructure.^25^ The ability to sustain high adherence rates and rational consumption patterns throughout the COVID-19 pandemic and the subsequent wartime period underscores the resilience of the implemented stewardship framework.^26,27^ In an environment characterized by resource constraints and external instability, the AMS program provided a stable scaffold for clinical decision-making, proving that quality improvement initiatives can function as a mechanism of institutional endurance.

Several limitations merit consideration. First, the use of dispensed units rather than DDD limits direct external benchmarking. However, internal validity remains robust: despite severe wartime fluctuations in absolute hospital throughput, normalizing consumption to major procedural volumes confirmed remarkably stable relative usage, validating raw units as a reliable proxy for structural formulary shifts.^28^ Second, as previously discussed, the reduction in length of stay is multifactorial and heavily influenced by broader secular trends rather than the AMS intervention alone.^23^ Nevertheless, the temporal alignment between the cessation of antibiotic orders and the reduction in hospitalization suggests a strong contributory effect. Finally, the absence of concurrent microbiological surveillance data precludes a direct assessment of the ecological impact of these prescribing changes on local resistance rates, an area prioritized for future research.

In conclusion, the implementation of a comprehensive antimicrobial stewardship program successfully dismantled entrenched broad-spectrum prescribing habits, shifting practice toward rational, guideline-concordant prophylaxis. This transition generated substantial clinical and economic benefits, primarily by eliminating unjustified postoperative administration and facilitating earlier patient discharge. Our findings validate the feasibility of administrative stewardship interventions, specifically “stop-orders” and dispensing restrictions, in transitional health systems. By relying on low-cost, protocol-based strategies rather than expensive infrastructure, this model demonstrates how healthcare facilities in high-pressure, resource-variable environments can maintain clinical quality and resilience. Future efforts should focus on integrating indication-based monitoring to refine the use of combination therapies and linking consumption data with resistance surveillance.

## Research transparency and reproducibility

The data are not publicly available due to institutional restrictions and confidentiality policies; aggregated data may be available from the corresponding author upon reasonable request.
